# Comparison of hydrocortisone 100 mg bolus plus 200 mg/day infusion *vs.* infusion alone in refractory septic shock

**DOI:** 10.1016/j.jointm.2025.02.002

**Published:** 2025-04-25

**Authors:** Ukrit Jiradechpitak, Theerapon Tangsuwanaruk, Patipan Sitthiprawiat, Borwon Wittayachamnankul

**Affiliations:** Department of Emergency Medicine, Faculty of Medicine, Chiang Mai University, Chiang Mai, Thailand

**Keywords:** Septic shock, Hydrocortisone, Intravenous infusion, Vasopressors, Adrenal insufficiency

## Abstract

**Background:**

Current guidelines recommend the addition of hydrocortisone 200 mg/day for the treatment of septic shock unresponsive to fluids and vasopressors. However, the benefits of adding a 100 mg bolus to this regimen remain unclear. The study assessed the efficacy of the administration of hydrocortisone 200 mg/day with or without a 100 mg bolus in refractory septic shock.

**Methods:**

This retrospective cohort study included adult patients with refractory septic shock treated at a tertiary care center between 2019 and 2023. Patients were divided into bolus group (receiving a 100 mg hydrocortisone bolus followed by a 200 mg/day continuous infusion) and non-bolus group (receiving a continuous infusion of 200 mg/day without the addition of a bolus) based on physician decision. The primary outcomes were the duration of vasopressor and shock reversal. Secondary outcomes included 28-day mortality and length of hospital stay. Comparisons between groups were performed using chi-squared tests, *t*-tests, and Kaplan–Meier survival analysis.

**Results:**

A total of 184 patients were included, 149 patients in the bolus group and 35 patients in the non-bolus group. The median vasopressor duration was 1 (interquartile range [IQR]: 1–2) days in both groups (*P*=0.967). Shock reversal occurred in 79.9 % of the bolus group and 82.9 % of the non-bolus group (OR=0.82, 95% CI: 0.30 to 2.23, *P*=0.688). Secondary outcomes in the bolus group and non-bolus group, including 28-day mortality (30.2 % *vs.* 22.9 %, OR=1.46, 95% CI: 0.62 to 3.43, *P*=0.745) and hospital length of stay (9 [IQR: 6-17] days *vs*. 11 [IQR: 5-15] days, *P*=0.875), did not show any significant differences. Kaplan–Meier survival analysis showed no difference in 28-day survival between groups (HR=1.29, 95% CI: 0.73 to 2.30, *P*=0.373).

**Conclusion:**

The addition of a 100 mg bolus to a 200 mg/day hydrocortisone regimen may not impact clinical outcomes in refractory septic shock.

## Introduction

Septic shock is one of the leading causes of mortality in critically ill patients, contributing significantly to the global burden of disease. Each year, sepsis affects nearly 48.9 million individuals worldwide, resulting in 11 million deaths, equating to approximately 20 % of total global mortality.^[^^1^^]^ Numerous studies support the use of corticosteroids to shorten the duration of hypotension in patients with refractory shock.^[^^2^^]^ In critically ill patients, adrenal insufficiency frequently co-occurs with septic shock, as both conditions share overlapping clinical presentations, including refractory hypotension and metabolic instability. This coincidence complicates diagnosis and highlights the need for effective treatments to address both conditions.^[^^3^^]^ Current guidelines, published by the Surviving Sepsis Campaign,^[^^4^^]^ recommend treatment with hydrocortisone at a dose of 200 mg/day for septic shock unresponsive to fluid resuscitation and vasopressors. This treatment has been shown to reduce vasopressor dependency and improve hemodynamic stability.^[^[5], [6]^]^ However, in real-world clinical practice, there are cases where hydrocortisone is administered as a 100 mg intravenous bolus, followed by a continuous infusion at a rate of 200 mg/day. This strategy is particularly useful for addressing adrenal insufficiency .^[^^7^^]^ However, the choice between treatment with the bolus plus infusion regimen or infusion alone remains controversial, with limited guidance provided by existing studies. Prior studies in acute respiratory distress syndrome (ARDS) and severe community-acquired pneumonia have suggested the potential benefits of hydrocortisone bolus in reducing inflammation and improving clinical outcomes.^[^[8], [9], [10]]

Previous studies indicate that increasing hydrocortisone from 200 mg to 300 mg daily does not improve shock reversal, mortality, or vasopressor duration. However, it may increase the risk of hyperglycemia and superinfections.^[^[11], [12]^]^ However, specific comparisons between the administration of 200 mg hydrocortisone with or without an initial 100 mg bolus remain largely unexplored. The objective of this study was to evaluate the efficacy and safety of the administration of hydrocortisone 200 mg/day with and without an initial 100 mg bolus in the management of refractory septic shock. Specifically, it focuses on clinical outcomes including shock reversal, duration of vasopressor use, 28-day mortality, and length of hospital stay.

## Methods

### Study design and setting

This is an observational retrospective cohort study conducted at Maharaj Nakorn Chiang Mai Hospital, a tertiary care hospital in Thailand. The study included patients treated in the emergency department between January 2019 and December 2023. Approval was received from the Institutional Review Board with a waiver of informed consent (registration number EME-2566-0453). Details related to all patients with missing data were removed before analysis.

### Participants

Inclusion criteria were patients aged 18 years or older who presented with septic shock, were treated with hydrocortisone in the emergency department, and were subsequently admitted. The diagnosis of sepsis or septic shock was based on the Third International Consensus Definitions for Sepsis and Septic Shock (SEPSIS-3).^[^^13^^]^ Specifically, patients who met the following criteria: sepsis was diagnosed with evidence of a sequential organ failure assessment (SOFA) score increased by two points from previous visits, if prior SOFA scores were unknown, a SOFA score ≥2 was used in conjunction with a confirmed source of infection. Septic shock was diagnosed as persistent hypotension requiring vasopressors to maintain a mean arterial pressure (MAP) of ≥65 mmHg, along with serum lactate levels >2 mmol/L, despite adequate fluid resuscitation. Patients were excluded if they declined full treatment, opted for palliative care, or had incomplete medical records, which prevented analysis.

### Data collection

Data for this study were collected retrospectively from electronic medical records. Patient demographics, including age, sex, weight, and comorbidities such as diabetes mellitus, hypertension, chronic kidney disease, and cancer, were recorded. Clinical information included the SOFA score, infection sites (*e.g.*, respiratory, urinary tract, intra-abdominal), baseline laboratory values specifically partial pressure of oxygen/fraction of inspired oxygen ratio, hemoglobin, neutrophil count, platelets, blood urea nitrogen, creatinine, bilirubin, and lactate levels. Microbiology results were also included. Details of management in the emergency department were collected, including initial intravenous fluid volume, defined as the amount of intravenous fluid administered before the initiation of vasopressor therapy, antibiotic regimens, and appropriateness of antibiotics, which was defined as the use of antibiotics that matched the sensitivity results from microbiological cultures. Other data collected included the use of mechanical ventilation and vasopressor therapy. Adverse events associated with corticosteroid therapy, such as hyperglycemia, secondary infections, and gastrointestinal bleeding, were inconsistently documented, limiting reliable assessment.

### Protocol

Patients were divided into two groups based on physician discretion, as no institutional protocol guided the choice of regimen. Bolus group included patients who received an initial 100 mg intravenous bolus of hydrocortisone, followed by a continuous infusion of 200 mg/day. Non-bolus group included patients who received a continuous infusion of hydrocortisone at 200 mg/day without a preceding bolus dose.

### Outcomes

The primary outcomes of the study were duration of vasopressor use (day), and shock reversal defined as the successful discontinuation of vasopressors for >24 h without recurrence of shock. Secondary outcomes were length of hospital stay and all-cause 28-day mortality in hospital.

### Sample size calculation

The sample size was calculated based on a 1:1 ratio between the intervention and control groups using data from previous studies.^[^^5^^]^ A mean time to resolution of shock of (5.00±5.19) days in the placebo group and (3.33±2.23) days in the hydrocortisone group was assumed, with a two-sided alpha error of 0.05 and power of 80 %. The required total sample size was determined as 180 participants (90 per group).

### Statistical analysis

Descriptive statistics were used to summarize baseline characteristics and clinical outcomes. Continuous variables were analyzed using mean values with standard deviations for normally distributed data and medians with interquartile ranges (IQRs) for non-normally distributed data. Comparisons between groups were performed using the independent *t*-test for normally distributed data and the Mann–Whitney *U* test for non-normally distributed data. Categorical variables were expressed as frequencies and percentages and analyzed using the chi-squares test or Fisher's exact test, as appropriate. Survival analysis was conducted to evaluate survival during the 28 days. Kaplan–Meier survival curves were generated, and differences between groups were assessed using the log-rank test. Binary outcomes were analyzed using logistic regression to calculate odds ratios with 95% confidence intervals. Survival analysis was conducted using Cox proportional hazards regression to derive hazard ratios (HR). All statistical analyses were performed using Stata® statistical software, version 17 (StataCorp LLC, College Station, TX, USA). A *P*-value of <0.05 was considered statistically significant.

## Results

### Baseline characteristics

A total of 184 patients with refractory septic shock were included, with 149 in the bolus group and 35 in the non-bolus group. There were no significant differences in the demographic and baseline characteristics (Table 1), with the exception of a higher prevalence of human immunodeficiency virus in the non-bolus group (5.7* %* *vs*. 0 %, *P*=0.003) and a greater frequency of intra-abdominal infections in the non-bolus group (31.4 % *vs.* 14.1 %, *P*=0.015). Primary adrenal insufficiency showed no significant difference between the bolus group and the non-bolus group (5.4 % *vs*. 2.9 %, *P*=0.535). Random cortisol levels indicative of Critical Illness-Related Corticosteroid Insufficiency (CIRCI) were higher in the bolus group (18.1 %) compared to the non-bolus group (5.7 %), but the difference was not statistically significant (*P*=0.064).

**Table 1:** Demographics, clinical characteristics, and baseline laboratory measurements of patients.

Characteristics	Non-bolus (*n* = 35)	Bolus (*n* = 149)	*P*-value
Baselines and demographics			
Age (years)	70.2 ± 15.4	69.3 ± 13.2	0.710
Female	13 (37.1)	73 (49.0)	0.206
Weight (kg)	53 (50–62)	53(48–63)	0.719
Comorbidities Diabetes mellitus	7 (20)	46 (30.9)	0.201
Hypertension	14 (40)	58 (38.9)	0.907
Chronic kidney disease	5 (14.3)	25 (16.8)	0.719
Congestive heart failure	1 (2.9)	11 (7.4)	0.329
Cancer	10 (28.6)	36 (24.2)	0.588
Human immunodeficiency virus	2 (5.7)	0 (0)	0.003
Primary adrenal insufficiency	1 (2.9)	8 (5.4)	0.535
Systemic lupus erythematosus	0 (0)	5 (3.4)	0.272
SOFA	6 (4–8)	7 (5–8)	0.123
Infection site Respiratory	14 (40)	49 (32.9)	0.425
Urinary tract	5 (14.3)	42 (28.2)	0.090
Skin/soft tissue	0 (0)	3 (2)	0.397
Intra-abdominal	11 (31.4)	21 (14.1)	0.015
Catheter	1 (2.9)	4 (2.7)	0.955
Other	4 (11.4)	30 (20.1)	0.232
Baseline laboratory measurement			
PaO_2_/FiO_2_ ratio	325.0 (270.5–435.0)	324.0 (257.0–428.0)	0.609
Complete blood count - Hemoglobin (g/dL)	10.5 (9.3–11.6)	9.8 (8.1–12.5)	0.467
Neutrophil count (×10^9^/L)	8.75 (5.99–15.47)	8.37 (4.24–13.89)	0.320
Platelets (×10^9^/L)	200 (133.5–290.5)	177 (110–253)	0.317
BUN (mg/dL)	26 (22–37)	31 (19–52)	0.409
Creatinine (mg/dL)	1.47 (1.12–2.33)	1.68 (1.18–2.90)	0.241
Bilirubin (mg/dL)	0.83 (0.55–1.095)	0.88 (0.48–2.23)	0.597
Random cortisol			0.064
<10 µg/dL*	2 (5.7)	27 (18.1)	
10–18 µg/dL†	3 (8.6)	24 (16.1)	
>18 µg/dL	30 (85.7)	98 (65.8)	
Lactate (mmol/L)	4.48 (3.03–6.55)	4.13 (2.65–7.01)	0.826
Positive culture	17 (48.6)	70 (47.0)	0.865
Gram-positive	13 (37.1)	51 (34.2)	0.745
Gram-negative	4 (11.4)	19 (12.8)	0.831

Data are presented as *n* (%) or median (interquartile range).

BUN: Blood urea nitrogen; FiO_2_: Fraction of inspired oxygen; PaO_2_: Partial pressure of oxygen; SOFA: Sequential organ failure assessment.

⁎ Random cortisol 10 µg/dL is a diagnostic criterion for critical illness-related corticosteriod insufficiency.^[^^14^^]^

† Random cortisol <18 µg/dL during stress suggests the diagnosis of adrenal insufficiency.

Management of shock in the emergency department was similar between the bolus and non-bolus groups (shown in Table 2). There were no significant differences in median intravenous fluid volumes initial antibiotic choices, or appropriateness of antibiotic therapy. Mechanical ventilation was required in 33.6 % of the bolus group and 28.6 % of the non-bolus group (*P*=0.571). Norepinephrine use was universal in both groups (*P*=1), with comparable doses (*P*=0.407). Adrenaline use and dosage were also similar (*P* = 0.830 and *P*=0.834). These findings indicate consistent shock management across both groups.

**Table 2:** Management of shock in the emergency room.

Characteristics	Non-bolus (*n*=35)	Bolus (*n*=149)	*P* value
Initial intravenous fluid management (mL)	1500 (1000–2000)	1500 (1100–2000)	0.803
Initial antibiotics Amoxicillin/clavulanate	1 (2.9)	3 (2.0)	0.758
3rd generation cephalosporin	11 (31.4)	48 (32.2)	0.929
Piperacillin/tazobactam	8 (22.9)	39 (26.2)	0.686
Carbapenem	15 (42.9)	60 (40.3)	0.779
Appropriateness of antibiotic therapy	13 (37.1)	44 (29.5)	0.381
Mechanical ventilation	10 (28.6)	50 (33.6)	0.571
Type and maximum dose of vasopressor Norepinephrine	35 (100)	149 (100)	1
Norepinephrine dose (μg/(kg·min))	0.45 (0.20–0.50)	0.25 (0.20–0.50)	0.407
Adrenaline	2 (5.71)	10 (6.71)	0.830
Adrenaline dose (μg/(kg·min))	1.1 (0.2–2.0)	0.6 (0.5–0.8)	0.834

Data are presented as *n* (%) or median (interquartile range).

### Primary outcomes

The outcomes of patients with refractory septic shock are presented in Table 3. Among the primary outcomes, including only surviving patients (*n*=148), the median duration of vasopressor use was 1 (IQR: 1–2) days in both the bolus and non-bolus groups, with no significant difference between the groups (*P*=0.967). Shock reversal rates were similar, occurred in 79.9 % of patients in the bolus group compared to 82.9 % in the non-bolus group (OR=0.82, 95% CI: 0.30 to 2.23, *P*=0.688).

**Table 3:** Outcomes of refractory septic shock patients treated with or without an initial 100 mg hydrocortisone bolus.

Characteristics	Non-bolus (*n*=35)	Bolus (*n*=149)	*P*-value
Vasopressor duration (day)*	1 (1–2)	1 (1–2)	0.967
Shock reversal	29 (82.9)	119 (79.9)	0.688
28-day all-cause mortality in hospital	8 (22.9)	45 (30.2)	0.745
Hospital stay (day)⁎	11 (5–15)	9 (6–17)	0.875

Data are presented as *n* (%) or median (interquartile range).

⁎ Including only surviving patients (*n* = 148).

### Secondary outcomes

**Figure 1:**
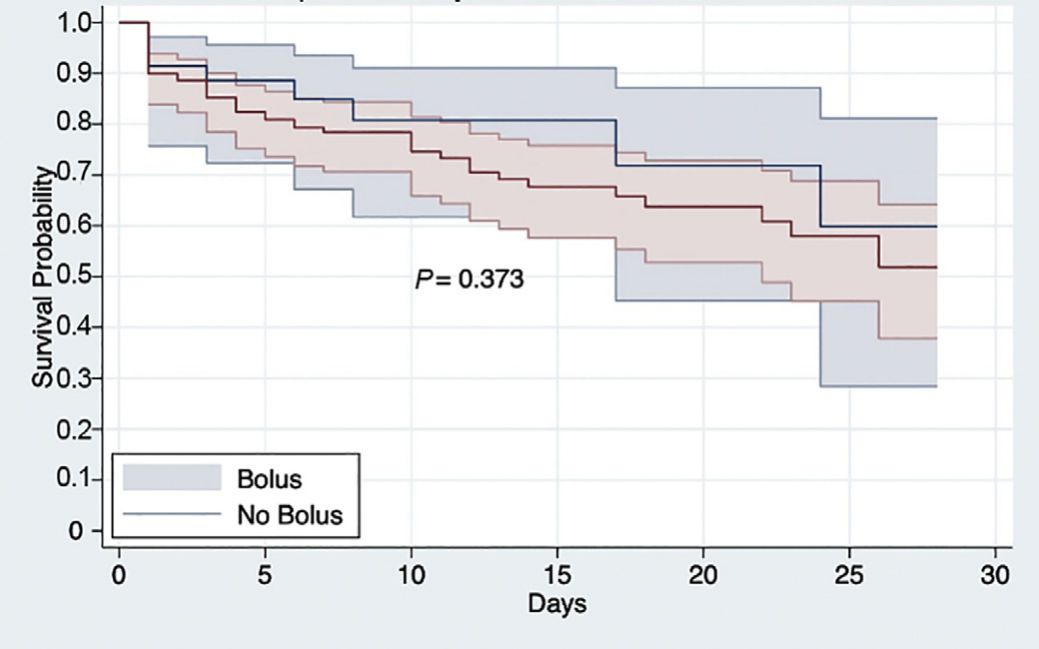
Kaplan–Meier survival curves comparing 28-day survival among septic shock patients receiving hydrocortisone 200 mg/day with or without an initial 100 mg bolus.

For secondary outcomes, the 28-day all-cause in-hospital mortality was slightly higher in the bolus group (30.2 %) compared to the non-bolus group (22.9 %), but this difference was not statistically significant (OR=1.46, 95% CI: 0.62 to 3.43, *P*=0.745). Kaplan–Meier survival analysis demonstrated no significant difference in survival between patients receiving hydrocortisone with a bolus dose and those receiving infusion alone (HR=1.29, 95% CI: 0.73 to 2.30, *P*=0.373, Figure 1). The survival curves and confidence intervals largely overlapped throughout the 28 days. Among surviving patients, the median hospital length of stay was 9 (IQR: 6–17) days in the bolus group and 11 (IQR: 5–15) days in the non-bolus group. The results showed no significant difference (*P*=0.875).

## Discussion

This study demonstrates that the addition of a 100 mg intravenous bolus to a 200 mg/day hydrocortisone infusion does not improve clinical outcomes in patients with refractory septic shock. Both regimens showed similar results in terms of vasopressor duration, shock reversal rates, 28-day mortality, and length of hospital stay. These findings suggest that bolus administration is unnecessary, and continuous infusion alone is sufficient to achieve hemodynamic stability.

Hydrocortisone administration accelerates shock resolution in patients with refractory septic shock, and we assumed that a higher dose might further enhance this effect. However, similar to results from previous research comparing 300 mg/day to the guideline-recommended 200 mg/day regimen,^[^[11], [12]^]^ our study found no significant difference in outcomes including shock reversal or vasopressor duration. The median vasopressor duration was 1 day in both groups. This shorter duration, compared to the 3-day median reported in previous studies,^[^^5^^]^ likely reflects early goal-directed therapy, effective source control, and prompt antibiotic initiation. Although earlier studies administered 300 mg/day continuously throughout the treatment period, our protocol differed by administering 300 mg hydrocortisone only on the first day, using a 100 mg bolus followed by a 200 mg/day infusion, and transitioning to 200 mg/day thereafter. Despite this variation, our findings were in alignment with the consensus that 200 mg/day is sufficient for the management of refractory septic shock without the need for higher doses or bolus administration, which is in alignment with all prior research findings.

In prior research, it has been found that a comparison between hydrocortisone and placebo has shown no significant differences in 28-day mortality or length of hospital stay between groups.^[^[5], [15]^]^ This supports the understanding that the main advantage of treatment with cortisone is in the achievement of hemodynamic stability rather than improving long-term outcomes. Therefore, the addition of a 100 mg bolus to the standard 200 mg/day regimen is unlikely to impact these outcomes and may represent an unnecessary component of treatment.

In clinical practice, two conditions that can mimic refractory septic shock are CIRCI and adrenal crisis, each with specific diagnostic criteria and treatment approaches. CIRCI is more commonly observed in critically ill patients with prolonged intensive care unit stays due to severe infections, organ failure, or sustained systemic inflammation.^[^^16^^]^ It is diagnosed based on a delta cortisol (response to adrenocorticotropic hormone [ACTH] stimulation) of ≤9 µg/dL or a random cortisol level <10 µg/dL, reflecting an inadequate adrenal response relative to critical illness. The recommended hydrocortisone dose for CIRCI is <400 mg/day, administered as a continuous infusion or in divided doses.^[^^14^^]^ Adrenal crisis, on the other hand, typically occurs in patients with primary adrenal insufficiency, such as those with Addison's disease or who are on chronic steroid therapy. It is characterized by acute cortisol deficiency, with random cortisol levels often <18 µg/dL during stress,^[^^17^^]^ along with refractory hypotension, hyperkalemia, or hyponatremia. Treatment requires an initial 100 mg bolus followed by a 200 mg/day infusion.^[^[7], [18]^]^ However, in our study, the number of patients who met the criteria for CIRCI or adrenal crisis was small, differing from previous studies that reported a higher incidence of CIRCI. For instance, one study reported an incidence of adrenal insufficiency in septic shock patients as high as 42 %.^[^^3^^]^ Although adrenal insufficiency, particularly CIRCI, is relatively common in septic shock, primary adrenal insufficiency is rare,^[^^19^^]^ and recommendations for endocrine emergencies may not be directly applicable. This discrepancy may be explained by the fact that previous studies included ACTH stimulation testing, whereas our study did not perform this test. This limitation underscores the importance of future research to stratify these patient groups more clearly and to consider the incorporation of ACTH testing for a more comprehensive evaluation. Although there was no difference between the two study groups, it remains uncertain whether a hydrocortisone bolus offers benefits in these patients.

### Limitation

A key limitation of this study is the inherent imbalance in sample size between the intervention and control groups, arising from its retrospective design and physician preference for hydrocortisone bolus use without a strict protocol. This imbalance may have introduced selection bias, potentially influencing the observed outcomes. Although a complete-case analysis was performed to reduce bias, the limited availability of comprehensive retrospective data for the non-bolus group remains a significant constraint. Although propensity score matching could have minimized baseline differences between groups, the limited sample size, particularly in the non-bolus group, precluded its application. In addition, the recording of vasopressor duration in hours, rather than days, could have provided more precise insights into potential differences between groups. Another limitation is that the study was conducted at a single center, which may limit the generalizability of the findings to other settings with different patient populations or treatment protocols. In addition, although hydrocortisone bolus has been explored in patients with severe community-acquired pneumonia and ARDS, our study did not consistently document these conditions. This precluded subgroup analysis and limits the applicability of findings to such populations. Future research should focus on conducting a randomized cross center controlled trial comparing 100 mg bolus and non-bolus groups while ensuring vasopressor duration is recorded in hours for greater precision. The study should also capture key complications associated with corticosteroid therapy, such as hyperglycemia, superinfection, and upper gastrointestinal hemorrhage, to provide a comprehensive safety and efficacy profile. In addition, patients with CIRCI and adrenal insufficiency should be excluded to ensure a more homogeneous study population and to better assess the effects of hydrocortisone in refractory septic shock.

## Conclusion

Our findings suggest that a bolus dose of hydrocortisone offers no significant clinical benefit compared to continuous infusion alone for refractory septic shock. These results highlight the sufficiency of guideline-recommended dosing and underscore the importance of individualized treatment approaches. Further research is warranted to definitively clarify the role, if any, of the administration of a hydrocortisone bolus in this patient population.

## CRediT authorship contribution statement

**Ukrit Jiradechpitak:** Writing – review & editing, Writing – original draft, Visualization, Methodology, Formal analysis, Data curation, Conceptualization. **Theerapon Tangsuwanaruk:** Software, Methodology, Investigation, Conceptualization. **Patipan Sitthiprawiat:** Visualization, Validation, Software, Methodology, Investigation, Formal analysis. **Borwon Wittayachamnankul:** Writing – review & editing, Writing – original draft, Validation, Supervision, Methodology, Funding acquisition, Formal analysis.
